# Ultrasound characteristics of primary fallopian tube carcinoma and the misdiagnosis and missed diagnosis

**DOI:** 10.12669/pjms.41.5.10600

**Published:** 2025-05

**Authors:** Dan Yan, Chunfang Shen, Rongrong Ru, Huijing Xu

**Affiliations:** 1 Dan Yan Department of Ultrasound, Affiliated Xiaoshan Hospital, Hangzhou Normal University, Hangzhou 311202, China; 2 Chunfang Shen Department of Obstetrics and Gynecology, Hangzhou 311202, China. Affiliated Xiaoshan Hospital, Hangzhou Normal University, Hangzhou 311202, China; 3 Rongrong Ru Department of Ultrasound, Affiliated Xiaoshan Hospital, Hangzhou Normal University, Hangzhou 311202, China; 4 Huijing Xu Department of Radiology, Hangzhou TCM Hospital Affiliated to Zhejiang Chinese Medical University, Hangzhou, 310007, China

**Keywords:** Primary fallopian tube carcinoma, Ultrasound, Characteristics, Misdiagnosis, Missed diagnosis

## Abstract

**Objective:**

This study retrospectively analyzed the ultrasound characteristics of primary fallopian tube carcinoma (PFTC) and the misdiagnosis and missed diagnosis.

**Methods:**

Data of 15 PFTC patients undergoing surgical treatment in Affiliated Xiaoshan Hospital, Hangzhou Normal University from August 2013 to September 2022 were collected. The clinical features, ultrasound characteristics, pathological diagnosis results and misdiagnosis and missed diagnosis by ultrasound were analyzed.

**Results:**

In 15 patients, there were 8 (53.33%) cases with vaginal bleeding, 6 (40.00%) cases with abdominal pain, five (33.33%) cases with pelvic mass, and two (13.33%) cases with vaginal discharge. There were 11 (73.33%) cases with CA125 level ≥ 35 U/ml. In 15 patients, 10 cases presented a sausage-shaped mass in adnexal region (type I PFTC) (two cases of cystic mass with papillary nodules, three cases of cystic-solid mass, five cases of hypoechoic or heterogeneous hypoechoic solid mass), four cases presented irregular hypoechoic mass in adnexal region (type II PFTC), and one case did not present the mass (type III PFTC). In 15 patients, three cases were accompanied by hydrosalpinx, two cases were accompanied by uterine fluid accumulation, and five cases were accompanied by abdominal or pelvic fluid accumulation. There were totally seven (46.66%) cases misdiagnosed or missed of diagnosis by ultrasound.

**Conclusion:**

The clinical manifestations of PFTC are diverse and lack of specificity. The ultrasound examination may have the misdiagnosis and missed diagnosis. PFTC should be highly suspected when there are characteristic ultrasound images including sausage-shaped mass companied by hydrosalpinx, uterine fluid accumulation, or abdominal or pelvic fluid accumulation. If the mass is small, it is prone to missed diagnosis.

## INTRODUCTION

Primary fallopian tube carcinoma (PFTC) is a very rare gynecological malignant tumor.^1^ PFTC is mostly unilateral in onset. Because the clinicopathological characteristics of PFTC are similar to serous epithelial ovarian cancer or primary serous peritoneal cancer, its true incidence may be underestimated. In the past decade, many studies^2^,^3^ have indicated a significant upward trend in the incidence of PFTC. PFTC is considered as one of the most difficult diagnostic malignant tumors due to its atypical clinical symptoms and lack of specific tumor markers. Although there are existing imaging methods such as computed tomography and magnetic resonance imaging (MRI), most PFTC cases are difficult to diagnose before surgery,^4^,^5^ and are prone to misdiagnosis. PFTC mainly relies on the intraoperative or postoperative pathological diagnosis, which inevitably leads to the insufficient preoperative preparation. Therefore, how to further improve the preoperative diagnostic rate of PFTC remains a challenge for clinical and imaging physicians.

Ultrasound is an effective method for detecting the lesions.^6^,^7^ In recent years, it is believed that the ultrasound examination has important value for the early diagnosis of PFTC. This may be due to the direct contact between transvaginal ultrasound probe and pelvic organs, which can display the fine structures of organs and tumors, as well as the hemodynamic characteristics of tumors.^8^ This study retrospectively analyzed the ultrasound characteristics of PFTC and the misdiagnosis and missed diagnosis, in order to further improve the ultrasound diagnosis accuracy of PFTC in clinical practice.

## METHODS

This study collected the data of 15 pathologically confirmed PFTC patients who underwent surgical treatment in Affiliated Xiaoshan Hospital, Hangzhou Normal University (Hangzhou, China) from August 2013 to September 2022. The age of patients was 33-74 years, with an average of 57.81±12.32 years. Among 15 patients, there were 10 (66.77%) postmenopausal women. The pregnancy frequency was 1-6 times, with parity frequency of 1-4 times and miscarriage frequency of 0-5 times. The clinical features included the irregular vaginal bleeding/discharge, abdominal pain and pelvic mass, with positive serum cancer antigen 125 (CA125) level. The ultrasound features included the adnexal area mass (cystic, cystic solid, solid), abdominal or pelvic fluid accumulation, and abdominal or pelvic organ and lymph node metastasist.

### Ethical Approval:

This study was approved by the ethics committee of Affiliated Xiaoshan Hospital, Hangzhou Normal University (No. 2024-025; Dated: July 22, 2024). Written informed consent was obtained from all participants.

### Ultrasound instruments:

ACUSON Sequoia ultrasound system (Siemens, Germany), iU22 xMATRIX ultrasound system (Philips, Netherlands) and Voluson E8 ultrasound system (GE HealthCare, USA) were adopted for the ultrasound examination. Their vaginal probe frequency was 4.0-9.0 MHz, 4.0-9.0 MHz and 5.0-9.0 MHz, respectively.

### Ultrasound diagnosis methods:

Patients were in the supine position and underwent the transvaginal ultrasound examination. Multiple sections were continuously scanned to observe the location, size, morphology, internal echo of abdominal and pelvic lesions and the proximity to surrounding organs. The color Doppler ultrasound was performed to observe the blood supply of lesions. If necessary, the abdominal ultrasound examination was performed to observe the tumor metastasis to other organs and lymph node in abdominal and pelvic cavities.

### Laboratory detection:

Venous blood was taken from the patients. After routine centrifugation, the serum was collected. The serum CA125 level was detected using the fully automatic electrochemiluminescence instrument. The serum CA125 level ≥ 35 IU/L was considered positive.

### Pathological examination:

Specimens of patients were fixed in 4% formaldehyde solution, and embedded in paraffin. The hematoxylin-eosin staining was performed. In addition, the immunohistochemical staining was conducted using the streptomycin antibiotin peroxidase method.

## RESULTS

### Clinical features:

Among 15 patients, there were eight (53.33%) cases with vaginal bleeding (mostly irregular and small amount of bleeding), six (40.00%) cases with abdominal pain (mostly dull pain), five (33.33%) cases with pelvic mass, and two (13.33%) cases with vaginal discharge. There were two cases of triple PFTC syndrome (vaginal bleeding/discharge+abdominal pain+pelvic mass) and five cases of binary PFTC syndrome (pelvic mass+vaginal bleeding/discharge or pelvic mass+abdominal pain), and two case of no clinical symptom (the pelvic mass was only detected by vaginal ultrasound during physical examination).

### Laboratory detection results:

Laboratory detection showed that, the serum CA125 level in 15 patients was 6.51-4398.72 U/ml, with average of 468.52±67.31 U/ml. There were 11 (73.33%) cases with CA125 level ≥ 35 U/ml.

### Ultrasonic characteristics:

**Fig.1:**
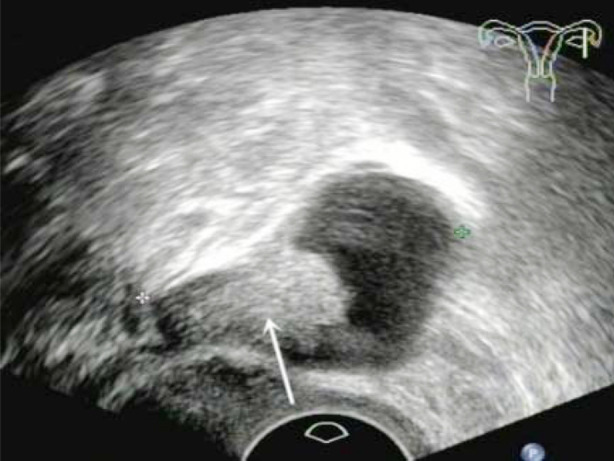
A patient with left type I PFTC (No. 2). The transvaginal two-dimensional ultrasound showed the sausage-shaped cystic mass with papillary nodules in left adnexal region (arrow).

**Fig.2:**
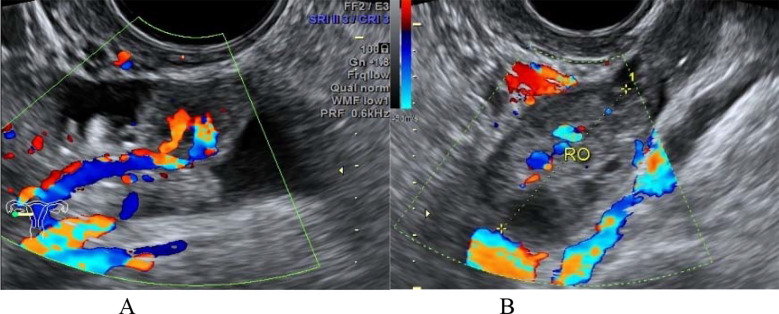
A patient with bilateral type I PFTC (No. 3). (A): the transvaginal color Doppler ultrasound showed the sausage-shaped cystic-solid mass in right paraovarian region; (B): the right normal ovary.

**Fig.3:**
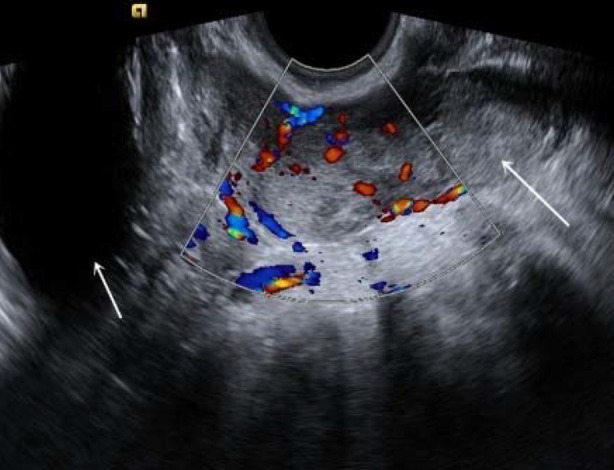
A patient with right type I PFTC (No. 9). The transvaginal color Doppler ultrasound showed the sausage-shaped hypoechoic solid mass in right adnexal region (long arrow), with rich blood flow signals and uterine fluid accumulation (short arrow) in flow imaging.

**Fig.4:**
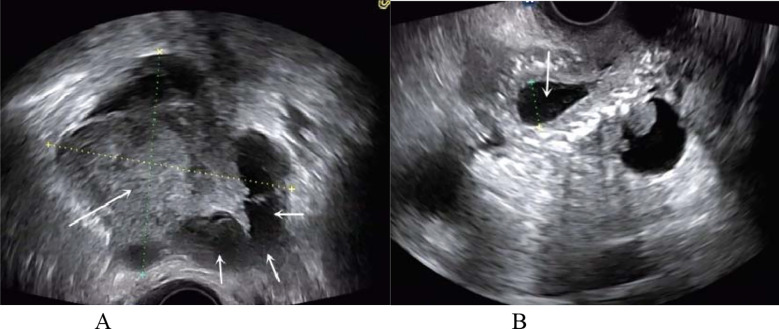
A patient with left type II PFTC (No. 14). (A): the transvaginal two-dimensional ultrasound showed the irregular and heterogeneous hypoechoic mass (long arrow) in the left adnexal region, with the dilated ureter (short arrow) adjacent to it; (B): the ultrasound showed the uterine fluid accumulation (arrow).

**Fig.5:**
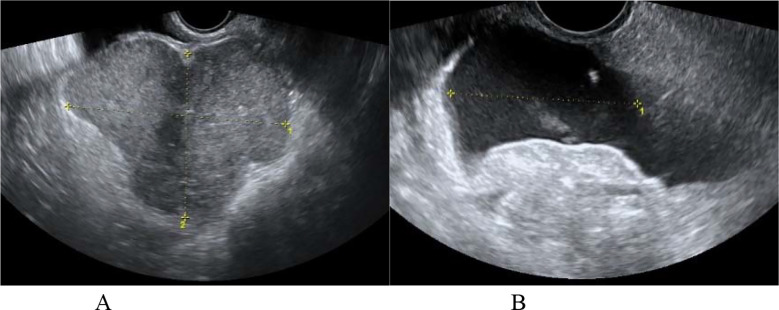
A patient with right type III PFTC (No. 15). (A): the transvaginal two-dimensional ultrasound showed the lobulated solid mass in left side of pelvic cavity, with no abnormal mass found in the right adnexal region; (B): the ultrasound showed the pelvic fluid accumulation.

Preoperative ultrasound examination showed that, in 15 patients there were 12 cases of unilateral adnexal mass (eight cases on left and four cases on right), two cases of bilateral adnexal masses, and one case with no adnexal mass. Among 15 patients, 10 cases presented a sausage-shaped mass (the mass grew along the fallopian tube, resembling sausage in appearance) in the adnexal region (type I PFTC) (two cases of cystic mass with papillary nodules (Fig.1), three cases of cystic-solid mass (Fig.2), five cases of hypoechoic or heterogeneous hypoechoic solid mass (Fig.3)), four cases presented irregular hypoechoic mass in adnexal region (type II PFTC) (Fig.4), and one case did not present the mass (type III PFTC) (Fig.5). The color Doppler ultrasound showed the detectable blood flow signals in tumor solid components or papillary nodules in 14 patients. In 15 patients, three cases were accompanied by hydrosalpinx, two cases were accompanied by uterine fluid accumulation, and two cases were accompanied by abdominal or pelvic fluid accumulation. The details of ultrasound characteristics in 15 patients were shown in Table-I.

**Table-I:** Ultrasound characteristics of 15 PFTC patients.

No.	Age	Tumor size (cm)	CA125 (IU/ml)	Site (adnexa)	Shape	Ultrasound finding	Type	Misdiagnosed	Missed of diagnosis
1	71	3.4	7.80	Bilateral	Sausage-shaped	Cystic mass with papillary nodules	I	-	-
2	47	3.0	21.11	Left	Sausage-shaped	Cystic mass with papillary nodules	I	-	-
3	49	4.4	36.22	Bilateral	Sausage-shaped	Cystic-solid mass	I	-	-
4	58	5.8	125.04	Left	Sausage-shaped	Cystic-solid mass	I	+	-
5	69	5.5	136.18	Left	Sausage-shaped	Cystic-solid mass	I	+	-
6	72	2.5	6.52	Right	Sausage-shaped	Hypoechoic solid mass	I	-	-
7	55	5.1	18.83	Left	Sausage-shaped	Heterogeneous hypoechoic solid mass	I	-	-
8	71	3.0	1089.05	Right	Sausage-shaped	Hypoechoic solid mass	I	-	-
9	74	6.1	56.22	Right	Sausage-shaped	Hypoechoic solid mass	I	-	-
10	48	4.5	38.20	Left	Sausage-shaped	Hypoechoic solid mass	I	-	-
11	49	3.8	103.27	Left	Irregular	Hypoechoic solid mass	II	+	-
12	49	7.8	773.42	Right	Irregular	Heterogeneous hypoechoic solid mass	II	+	-
13	33	6.0	74.93	Left	Irregular	Hypoechoic solid mass	II	+	-
14	68	7.2	133.82	Left	Irregular	Heterogeneous hypoechoic solid mass	II	+	-
15	54	*	4398.59	Right	*	No mass found	III	-	+

* No data.

### Pathological diagnosis results:

**Fig.6:**
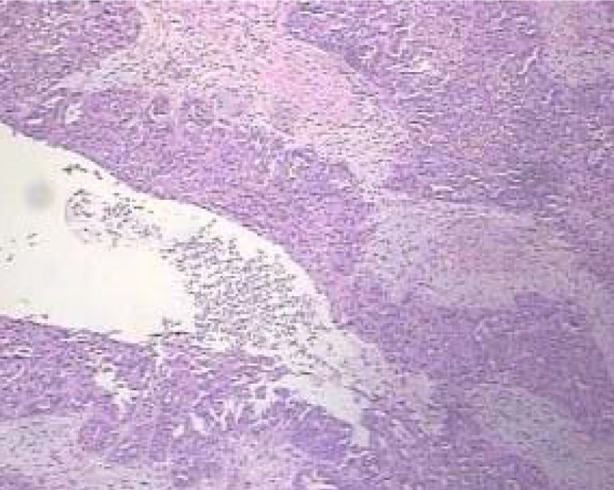
A patient with right type III PFTC (No. 15). The pathology showed that the tumor cells were arranged in glandular, papillary, or solid pattern. The tumor tissue formed papillary or glandular tubular structures, and partial areas were diffuse and patchy, with multiple nuclear fissions and invasion of tube wall (HE × 100).

Pathological diagnosis confirmed that all 15 patients were with PFTC. Under the microscope, the tumor cells were arranged in glandular, papillary, or solid pattern. The tumor tissue formed papillary or glandular tubular structures, and partial areas were diffuse and patchy, with multiple nuclear fissions and invasion of tube wall (Fig.6). There were five cases of pelvic implant metastasis, five cases of pelvic lymph node metastasis, three cases of retroperitoneal lymph node metastasis, and one case of inguinal lymph node metastasis.

### Preoperative misdiagnosis and missed diagnosis by ultrasound:

Among 10 cases of type I PFTC, 8 (53.33%) cases were preoperative diagnosed with PFTC by ultrasound, one case was misdiagnosed as ovarian cancer due to cystic solid mass, and one case was misdiagnosed as uterine fibroid. All four cases of type II PFTC were misdiagnosed as ovarian cancer. One case of type III PFTC was with missed diagnosis. There were totally seven (46.66%) cases misdiagnosed or missed of diagnosis.

## DISCUSSION

This study retrospectively analyzed the ultrasound characteristics of PFTC and the misdiagnosis and missed diagnosis. Results showed that, In 15 PFTC patients, there were 8 (53.33%) cases with vaginal bleeding, 6 (40.00%) cases with abdominal pain, five (33.33%) cases with pelvic mass, and two (13.33%) cases with vaginal discharge. There were 11 (73.33%) cases with CA125 level ≥ 35 U/ml. In 15 patients, 10 cases presented a sausage-shaped mass in adnexal region (type I PFTC) (two cases of cystic mass with papillary nodules, three cases of cystic-solid mass, five cases of hypoechoic or heterogeneous hypoechoic solid mass), four cases presented irregular hypoechoic mass in adnexal region (type II PFTC), and one case did not present the mass (type III PFTC). In 15 patients, three cases were accompanied by hydrosalpinx, two cases were accompanied by uterine fluid accumulation, and five cases were accompanied by abdominal or pelvic fluid accumulation. There were totally seven (46.66%) cases misdiagnosed or missed of diagnosis by ultrasound.

PFTC often occurs in women aged 40-65 years, and is more common in postmenopausal women, but it can also occur in young women.^9^ In this study, the average age of 15 patients was 57.81±12.32 years, and there were 10 (66.67%) menopausal patients, which was consistent with previous report. There were two cases of triple PFTC syndrome and four cases of binary PFTC syndrome. Although the triple PFTC syndrome has high specificity for diagnosing PFTC, less than 10% of patients exhibit typical triads.^10^ It is worth noting that, for PFTC patients with abnormal vaginal bleeding, 75% of them are preoperatively misdiagnosed as endometrial cancer. This also suggests that the ultrasound examination is particularly important when clinical symptoms are atypical. In this study, 11 cases had elevated serum CA125 level. The elevated CA125 level is helpful for the diagnosis of PFTC, but it exists in relatively common types such as ovarian cancer, fallopian tube and ovarian abscess.^11^ Therefore, the comprehensive analysis in combination with other clinical and imaging examinations is necessary.

According to different manifestations, the diagnostic efficacy of ultrasound for PFTC varies greatly. In this study, the ultrasound manifestations of mass in 15 PFTC patients were divided into three types including type I, II and III. In this study, 10, four and one cases presented the type I, II and III mass, respectively. Pathological basis of type I PFTC may be that the tumor mostly originates from the ampulla mucosa of fallopian tubes, with thickened tube wall and formation of intraluminal mass that blocks the fallopian tube and causes the tubal dilation.^12^ Therefore, it can produce a characteristic appearance of a sausage-shaped mass on ultrasound image.

In this study, among 10 cases of type I PFTC, eight cases were preoperative diagnosed with PFTC. One case was misdiagnosed as ovarian cancer. The reason of misdiagnosis may be due to the lack of experience of the examining physician, who cannot recognize the sausage-shaped appearance of mass. In addition, one case was misdiagnosed as uterine fibroid. The reason of misdiagnosis may be due to its large size and proximity to uterus, without pelvic fluid accumulation, pelvic peritoneal implantation, or lymph node metastasis. However, by careful analysis, PFTC and uterine fibroid can be distinguished, because the PFTC grows along the fallopian tube, forming a sausage-shaped mass, while the uterine fibroid is generally round in shape.

Type II PFTC may form based on type I PFTC. As the mass progresses, it destroys the structure of fallopian tube, penetrates the serosa, and invades outward, forming the irregular mass. At this time, the mass may be accompanied by pelvic peritoneum implantation or pelvic fluid accumulation. PFTC and ovarian cancer have similar clinical characteristics, and the ultrasound examination is difficult to distinguish them. In this study, all four cases of type II PFTC were misdiagnosed as ovarian cancer before surgery. However, a retrospective analysis has revealed that there are still distinguishing points between ultrasound images of PFTC and ovarian cancer. The PFTC may be accompanied by hydrosalpinx and uterine cavity effusion, while the ovarian cancer did not.

Pathological basis of type III PFTC is that the tumor occurs at the fimbriae end. This type of tumor has a small mass that does not form the hydrosalpinx, and the ultrasound examination is easily affected by intestinal gas interference. Therefore, the ultrasound examination cannot detect the sausage-shaped mass, which is prone to missed diagnosis. In this study, one case of type III PFTC was with missed diagnosis. Therefore, for type III PFTC, if the PFTC is clinically suspected, but the ultrasound does not detect the mass in adnexal region, the MRI examination should be performed to avoid the missed diagnosis.^13^

It is reported that the adnexal mass with hydrosalpinx and uterine fluid accumulation are beneficial for the differential diagnosis of PFTC.^5^ In this study, there were three cases of hydrosalpinx, two cases of uterine fluid accumulation, and five cases of abdominal or pelvic fluid accumulation, which is consistent with previous report. We believe that, when there is no typical feature of sausage-shaped mass in adnexal region, but the hydrosalpinx, uterine fluid accumulation, or abdominal or pelvic fluid accumulation appears, the possibility of PFTC should be considered.

In view of the results and our experiences, this study has provided an important basis for further improving the ultrasound diagnosis accuracy of PFTC, and has obvious clinical significance. However, due to the limited sample size, the findings of this study needs to be further verified by more cases in the next research.

### Limitations:

The sample size of this study was relatively small. If the sample size can be further expanded, the conclusion may be more convincing.

## CONCLUSION

The incidence of PFTC is low, and its clinical manifestations are diverse and lack of specificity. The ultrasound examination may have misdiagnosis and missed diagnosis. PFTC should be highly suspected when there are characteristic ultrasound images including sausage-shaped mass companied by hydrosalpinx, uterine fluid accumulation, or abdominal or pelvic fluid accumulation. If the mass is small, it is prone to missed diagnosis, and other imaging examinations should be performed.

### Authors’ Contributions:

**DY** and **CS:** Conceived and designed the study. Literature search. **RR:** Collected the data and performed the analysis. Critical Review. **HX:** Was involved in the writing of the manuscript and is responsible and accountable for the accuracy or integrity of the work.

## References

[1] Maeda M, Hisa T, Matsuzaki S, Ohe S, Nagata S, Lee M (2022). Primary Fallopian tube carcinoma presenting with a massive inguinal tumor:a case report and literature review. Medicina (Kaunas).

[2] Ma Z, Gao L, Li H, Li J, Zhang G, Xue Y (2021). Clinical characteristics of primary Fallopian tube carcinoma:A single-institution retrospective study of 57 cases. Int J Gynaecol Obstet.

[3] Thanasa E, Stamouli D, Gerokostas EE, Balafa K, Koutalia N, Thanasas I (2022). Primary fallopian tube carcinoma:an extremely rare gynecological cancer misdiagnosed intraoperatively as benign ovarian neoplasm:a case report. Clin Pract.

[4] Dai N, Deng S, Yang Y, Sang S (2022). 18-F fluorodeoxyglucose positron emission tomography/computed tomography findings of bilateral primary fallopian tube carcinoma and metastasis to the uterus:a case report and literature review. J Int Med Res.

[5] Yang Y, Xiao Z, Liu Z, Lv F (2020). MRI can be used to differentiate between primary fallopian tube carcinoma and epithelial ovarian cancer. Clin Radiol.

[6] Zhang H, Wang J, Guo R (2020). Application value of color Doppler ultrasound and ultrasound contrast in the differential diagnosis of ovarian tumor. Pak J Med Sci.

[7] Xu S, He H, Jiang M (2023). Comparison of the effects of contrast-enhanced ultrasound and conventional ultrasound-guided radiofrequency ablation on benign thyroid nodules. Pak J Med Sci.

[8] Kim EB, Lee TH, Kim JS, Choi IH (2014). Primary fallopian tube carcinoma diagnosed with endoscopic ultrasound elastography with fine needle biopsy. Clin Endosc.

[9] Vasiljevic M, Pazin V, Dzatic O, Jeremic D (2007). Primary fallopian tube carcinoma in a 51-year-old postmenopausal woman-case report. Eur J Gynaecol Oncol.

[10] Alvarado-Cabrero I, Young RH, Vamvakas EC, Scully RE (1999). Carcinoma of the fallopian tube:a clinicopathological study of 105 cases with observations on staging and prognostic factors. Gynecol Oncol.

[11] Li S, Yu M, Bai W, Shi J, Di W (2021). Long-term follow-up of 46 cases of primary fallopian tube carcinoma:a single institute study. Ann Palliat Med.

[12] Bachert SE, McDowell A, Piecoro D, Baldwin Branch L (2020). Serous tubal intraepithelial carcinoma:a concise review for the practicing pathologist and clinician. Diagnostics (Basel).

[13] Lee SI, Kang SK (2023). MRI Improves the Characterization of Incidental Adnexal Masses Detected at Sonography. Radiology.

